# Artificial Intelligence in Kidney Disease: A Comprehensive Study and Directions for Future Research

**DOI:** 10.3390/diagnostics14040397

**Published:** 2024-02-12

**Authors:** Chieh-Chen Wu, Md. Mohaimenul Islam, Tahmina Nasrin Poly, Yung-Ching Weng

**Affiliations:** 1 Department of Healthcare Information and Management, School of Health and Medical Engineering, Ming Chuan University, Taipei 111, Taiwan; drluiswu@gmail.com; 2 Outcomes and Translational Sciences, College of Pharmacy, The Ohio State University, Columbus, OH 43210, USA; d610106004@tmu.edu.tw; 3 Graduate Institute of Biomedical Informatics, College of Medical Science and Technology, Taipei Medical University, Taipei 110, Taiwan; d610108004@tmu.edu.tw

**Keywords:** artificial intelligence, machine learning, deep learning, kidney disease, bibliometric study

## Abstract

Artificial intelligence (AI) has emerged as a promising tool in the field of healthcare, with an increasing number of research articles evaluating its applications in the domain of kidney disease. To comprehend the evolving landscape of AI research in kidney disease, a bibliometric analysis is essential. The purposes of this study are to systematically analyze and quantify the scientific output, research trends, and collaborative networks in the application of AI to kidney disease. This study collected AI-related articles published between 2012 and 20 November 2023 from the Web of Science. Descriptive analyses of research trends in the application of AI in kidney disease were used to determine the growth rate of publications by authors, journals, institutions, and countries. Visualization network maps of country collaborations and author-provided keyword co-occurrences were generated to show the hotspots and research trends in AI research on kidney disease. The initial search yielded 673 articles, of which 631 were included in the analyses. Our findings reveal a noteworthy exponential growth trend in the annual publications of AI applications in kidney disease. *Nephrology Dialysis Transplantation* emerged as the leading publisher, accounting for 4.12% (26 out of 631 papers), followed by *the American Journal of Transplantation* at 3.01% (19/631) and *Scientific Reports* at 2.69% (17/631). The primary contributors were predominantly from the United States (*n* = 164, 25.99%), followed by China (*n* = 156, 24.72%) and India (*n* = 62, 9.83%). In terms of institutions, Mayo Clinic led with 27 contributions (4.27%), while Harvard University (*n* = 19, 3.01%) and Sun Yat-Sen University (*n* = 16, 2.53%) secured the second and third positions, respectively. This study summarized AI research trends in the field of kidney disease through statistical analysis and network visualization. The findings show that the field of AI in kidney disease is dynamic and rapidly progressing and provides valuable information for recognizing emerging patterns, technological shifts, and interdisciplinary collaborations that contribute to the advancement of knowledge in this critical domain.

## 1. Introduction

Kidney disease remains a global public health concern due to its higher prevalence and rising incidence [1,2]. Existing challenges related to tackling the burden of kidney disease include late-stage diagnoses, limited treatment options for end-stage kidney disease (ESKD), and disparities in access to healthcare [3,4,5]. The asymptomatic nature of the disease in its initial stages often hinder early detection, leading to delayed interventions and higher mortality rates [6]. Individuals with ESKD have limited alternatives, primarily dialysis or transplantation. However, access to these options is hampered by geographic, financial, and organ availability constraints [7,8]. Prior research emphasized the critical need to promptly tackle these challenges by identifying patients in earlier stages [4,9]. Artificial intelligence (AI) has emerged as a revolutionary tool in healthcare, particularly in the early prediction of kidney disease [10]. AI technologies, including machine learning algorithms, have shown substantial capabilities in analyzing diverse sets of patient data, encompassing clinical records, imaging, and genetic information. Numerous studies have been conducted leveraging these models to predict the onset of kidney disease at early stages [11,12], which facilitates timely interventions and improved patient outcomes [13]. The higher accuracy of these models has a potential impact on reducing the burden of kidney disease. Integration of AI tools into real-world clinical practices represents a promising frontier in proactive healthcare management, offering novel approaches to predict and mitigate the impact of kidney disease at its earliest stages [14,15]. Given the dynamic and rapidly evolving nature of AI in kidney disease research, a bibliometric analysis proves invaluable in comprehensively assessing research trends over time. This method facilitates an objective evaluation of both the quantity and quality of research outputs, unveiling key thematic areas, influential authors, and impactful journals [16,17]. Through the application of bibliometric analysis, we gain insights into the most frequently explored topics at the intersection of AI and kidney disease, thereby assisting in the identification of research gaps and areas needed for further exploration. Additionally, understanding the global landscape of AI research in kidney disease is vital for fostering international collaborations and knowledge exchange. Therefore, this study aims to offer an up-to-date and extensive overview of AI research in the realm of kidney disease through the application of bibliometric analysis.

## 2. Methods

### 2.1. Search Strategy

We collected relevant articles from the Web of Science (WoS) Core Collection by Clarivate Analytics in the USA, as it offers a wide range of bibliometric indicators and encompasses literature from various disciplines [17,18,19]. We formulated our search strategy by screening search terms found in previously published articles and consulting with experts who have conducted bibliometric studies. The conclusive search was conducted on 20 November 2023, ensuring the inclusion of all relevant articles published between 1 January 2010 and 20 November 2023. We used search terms associated with (1) kidney disease and (2) artificial intelligence models, combining the terms using Boolean Operators (“OR”, “AND”) (Supplementary Table S1).

### 2.2. Inclusion and Exclusion

All journal articles related to the application of AI models in kidney disease were subject to screening. Articles were considered for inclusion in final analysis if they met the following criteria: (1) written in the English language, (2) focused on kidney disease, and (3) involved AI models. Given the dynamic nature of AI research with frequent updates, we included research or review articles from peer-reviewed journals, conference proceedings, and early access publications. However, editorial materials, book chapters, and books were excluded from the bibliometric analysis.

### 2.3. Screening Strategy

Two authors conducted an independent screening of titles and abstracts from the collected articles and verified their validity. Any disagreements at this stage were resolved through discussions. Finally, data from the selected articles were gathered and stored in **.txt formats.

### 2.4. Bibliometric Analysis

This study seeks to explore the following inquiries, aiming to build upon previous research on the utilization of AI in kidney diseases:

1. Which countries, institutions, sources, and authors exhibit the highest productivity in the field of AI applied to kidney disease?
2. What are the prominent research topics and themes in the application of AI to kidney disease?
3. What methods and keywords are mainly used in the existing body of literature?

#### 2.4.1. Growth Rate of Publications

The calculation of the annual growth rate of publications involved determining the annual publication count, annual growth, and average growth rate of publications, employing the following methods:

(1) $$Annual\;growth=\left(Total\;number\;of\;articles\;in\;current\;year\right)-\left(Total\;number\;of\;articles\;in\;previous\;year\right)\;$$

(2) $$Average\;growht\;rate=\frac{\left[(N-N_{k-1})\right]}{N_{k-1}}\times 100$$

where N is the total number of articles in the current year and $N_{k-1}$ is the total number articles in the previous year.

#### 2.4.2. Publication Productivity

We analyzed publication trends, emphasizing the most prolific journals, countries, institutions, and authors. Specifically, we considered the top 10 most productive journals with categories, the top 10 most prolific countries with economic status, and the top 10 authors and institutions with countries. Rankings for countries, journals, institutions, and authors were determined based on the respective number of published articles.

#### 2.4.3. Research Hot Spot Tendencies

The VOSviewer software (Version 1.6.20) from the Centre for Science and Technology Studies at Leiden University was used to create a network map and clusters based on publications from 2010 to 2023. Network maps for regions/countries, institutions, and keywords were generated and presented in various clusters. Each node in the network map is denoted by a labeled circle, with larger circles signifying higher frequencies. The color of each circle corresponds to its respective cluster, while the strength of associations between nodes is conveyed through the thickness and length of links.

## 3. Results

### 3.1. Search Findings

**Figure 1:**
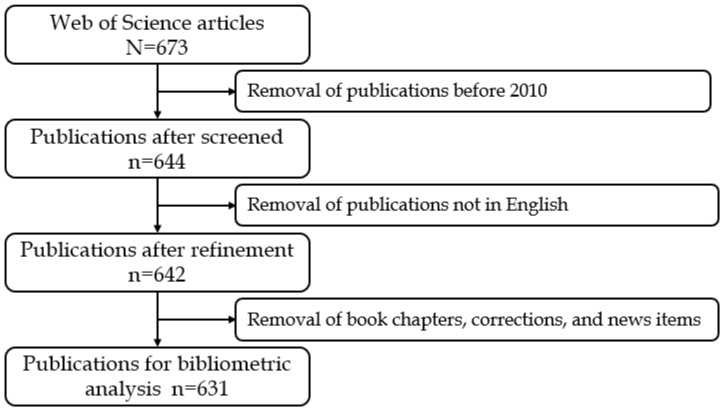
Screening flowchart for the application of artificial intelligence to kidney disease research.

Figure 1 illustrates the flow of the search and screening process. The initial search yielded 673 articles, and after excluding 31 based on pre-defined inclusion criteria, the screening phase involved 642 articles. Ultimately, the final bibliometric analysis comprised 621 articles.

### 3.2. Overall Trends

**Figure 2:**
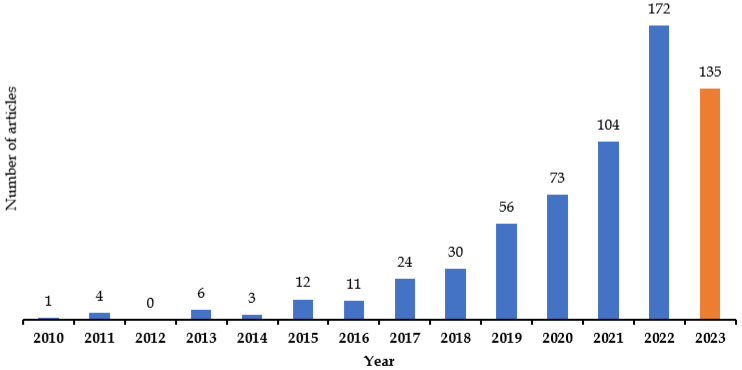
Publication outputs from 2010 to 2023 (Orange color bar represent partial year).

The number of annual publications on the application of AI in the domain of kidney disease increased from one article in 2010 to one hundred and seventy-two articles in 2022 (Figure 2). Before 2015, the number of annual articles did not reach 10. The average annual growth rate of articles was a maximum of 65.38% in 2022 and showed an 8.33% decline in 2016 (Table 1).

### 3.3. Journals and Their Subject Categories

Overall, the papers were published by 324 different journals. As Table 2 shows, the *Journal of Nephrology Dialysis Transplantation* published the most papers (4.12%, 26/631 papers) followed by *American Journal of Transplantation* (3.01%, 19/631) and *Scientific Reports* (2.69%, 17/631).

### 3.4. Distribution of Source Countries/Regions

Examining the origin of research often involves considering the country of the corresponding author. In our analysis, we found authors representing 68 countries. The preeminent contributors were from the United States (*n* = 164, 25.99% of all published articles), followed by China (*n* = 156, 24.72%) and India (*n* = 62, 9.83%). Notably, a substantial majority of published articles, 94.13% (*n* = 594) originated from researchers based in these top ten countries (Table 3).

**Figure 3:**
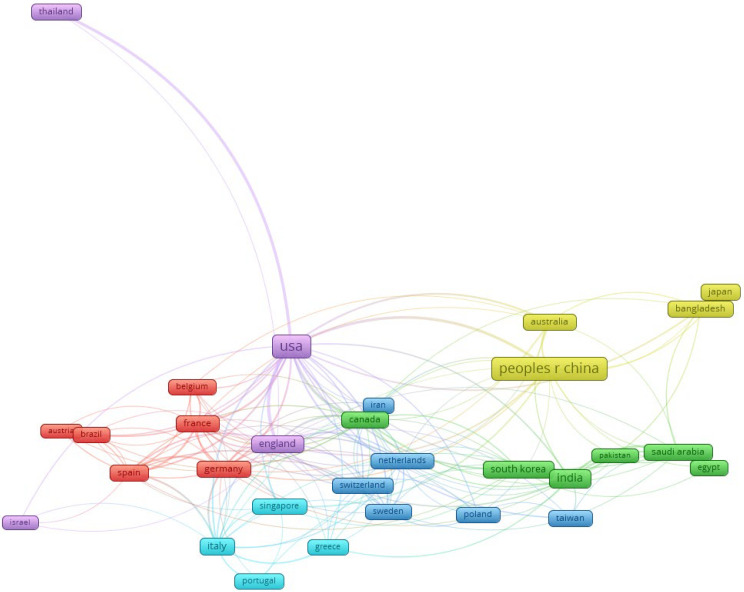
The network map among countries focusing on the application of artificial intelligence research in kidney disease, 2010–2023.

Figure 3 Illustrates the co-authorship analysis involving countries that published a minimum of five articles. The analysis reveals a total of six clusters, identified by distinct colors. For example: both cluster 1 (red color) and cluster 2 (green color), comprising six countries.

### 3.5. Distribution of Institutions

Based on our study findings, 1252 institutes actively contributed to at least one research article. Table 4 presents the top 10 research institutions that demonstrated notable productivity in the application of AI to kidney disease. Mayo Clinic led the list with 27 articles, followed by Harvard University with 19 articles, Sun Yat-Sen University with 16 articles, and Sichuan University with 15 articles.

**Figure 4:**
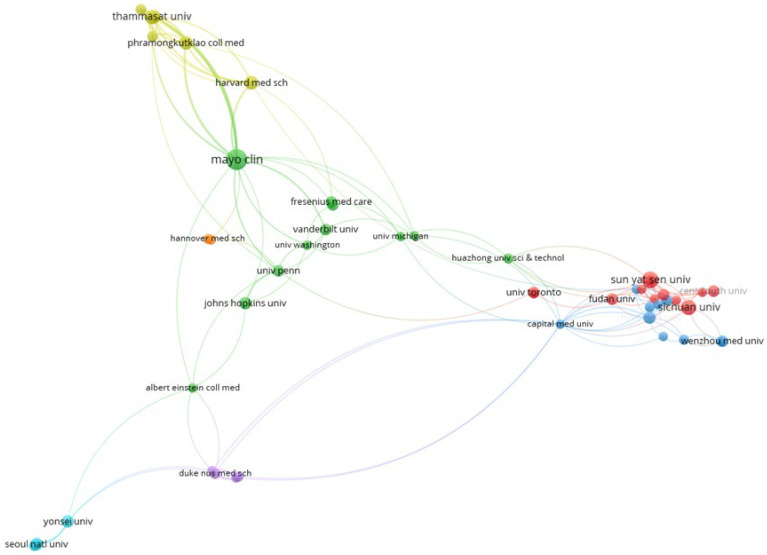
The network map among institutions focusing on the application of artificial intelligence research in kidney disease, 2010–2023.

Figure 4 illustrates the co-authorship analysis involving 58 institutions that have published a minimum of five articles. The analysis reveals a total of seven clusters, identified by distinct colors (cluster 1 in red, comprising twelve institutions; cluster 2 in green, including eleven institutions; and cluster 7 in aqua color, comprising two institutions).

### 3.6. Authors

In total, 3775 authors contributed to the 631 articles. The top 10 authors (Table 5) contributed to 124 articles (19.65% of all articles). Cheungpasitporn W. contributed to most papers (*n* = 16) followed by Thongprayoon C (*n* = 16), Leeaphorn N (*n* = 13), and Cooper M (*n* = 12).

### 3.7. Keywords

**Figure 5:**
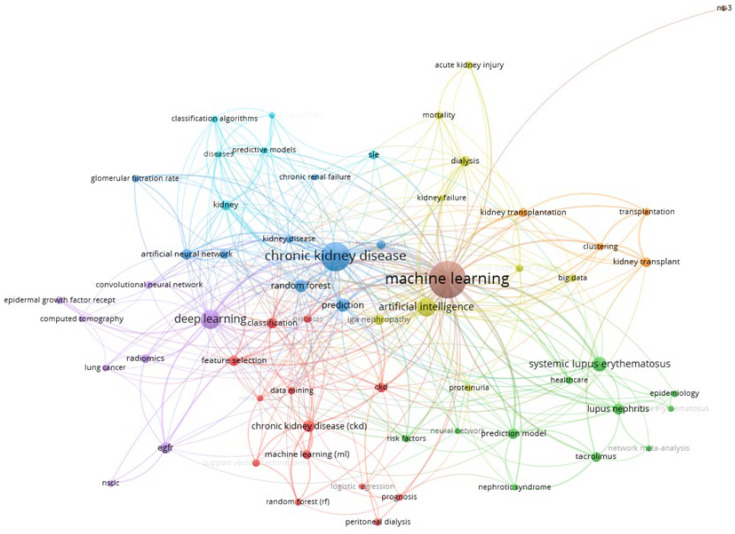
The co-occurrence network of the top 65 keywords in the application of AI in kidney disease research, 2010–2023.

A total of 1139 keywords were employed in these studies, and the top 65 keywords were classified into five clusters through keyword-clustering analysis (Figure 5). The five most prevalent keywords included: (a) AI terms: machine learning (*n* = 169), deep learning (*n* = 47), artificial intelligence (*n* = 44), random forest (*n* = 20), and artificial neural network (*n* = 14); (b) diseases: chronic kidney/CKD (*n* = 132), lupus nephritis (*n* = 16), kidney transplant (*n* = 12), acute kidney injury (*n* = 8), and end-stage renal disease (*n* = 7); (c) process terms: prediction/prediction model (*n* = 33), classification (*n* = 14), clustering (*n* = 8), prognosis (*n* = 6), and diagnosis (*n* = 4).

### 3.8. Top Cited Articles

We investigated the utilization of AI in the realm of kidney disease research. In Table 6, we present the ten most cited articles, garnering a combined total of 683 citations, which were published from 2017 to 2021. The publication with the highest citation count, titled “*Neural network and support vector machine for the prediction of chronic kidney disease: A comparative study*”, which was published in *Computers in Biology and Medicine* in 2019, has amassed 104 citations as of 20 November 2023.

## 4. Discussion

Artificial intelligence (AI) has become a key driving force in the realm of kidney disease, making substantial contributions to its diagnosis, prognosis, and overall management. Nowadays, AI models leverage extensive datasets comprising patient records, imaging studies, and genetic information, demonstrating remarkable performance in predicting the early onset of kidney disease [30,31]. Furthermore, diagnostic tools developed by AI models enhance precision and efficiency in identifying renal abnormalities, thereby facilitating timely interventions. Through bibliometric analysis, this study identified and examined 631 articles focusing on the application of AI in kidney diseases. The analysis unveiled prevalent themes, influential authors, and high-impact journals, providing insights into the most frequently explored topics at the intersection of AI and kidney disease. There has been a noticeable increase in the number of articles focusing on the application of AI in kidney disease, especially after the year 2016. This study also used clustering algorithms to group nodes that share strong connections, creating distinct clusters within the network. The strength of the connections was determined by co-authorship frequency, co-citation strength, or keyword co-occurrence. These clusters are visually represented in varying colors, and the size of nodes acts as an indicator of the significance or centrality of entities within their respective clusters. These findings may help researchers to delve into these clusters, pinpoint influential nodes, and give valuable insights into thematic concentrations and collaborative patterns in the literature.

Our study found that the predominant contributors were from the United States, followed by China and India. Remarkably, nearly 95% of the published articles were authored by researchers from the top ten countries. Furthermore, a higher percentage of articles originated from developed nations (categorized by the World Bank) [32,33]. Notably, institutions in developed countries actively conducted research on the application of AI in the context of kidney disease [34]. Recently, low-income and developing countries have focused on AI research for disease management, addressing healthcare challenges more effectively [35]. However, researchers in these countries often encounter resource shortages and limited access to specialized medical expertise when developing AI models [36,37]. Despite these challenges, these nations are on their way to developing advance AI tools because automated diagnosis, patient monitoring, and treatment planning prove to be cost-effective. Such advancements hold the potential to narrow the healthcare disparity gap by providing scalable, accessible, and efficient tools. Additionally, AI enables the optimization of limited resources, offering more precise and personalized healthcare interventions [38,39]. Through a comprehensive effort in AI research, low-income countries can leverage innovative technologies to enhance early detection, improve patient outcomes, and establish sustainable strategies for managing kidney diseases within resource-constrained settings.

The *Journal of Nephrology Dialysis Transplantation* led in the number of published papers, followed by the *American Journal of Transplantation* and *Scientific Reports*. Additionally, it is worth highlighting that a significant proportion of articles were published in open-access journals and those with high impact factors. Research indicates that open-access journals generally receive higher citation rates compared to subscription-based journals due to their high accessibility and visibility [40,41]. Nowadays, researchers are increasingly opting for open-access platforms, enabling their work to reach a wider audience, including researchers, practitioners, and the public, particularly in low-income countries. The practice of freely sharing scholarly works not only facilitates broader knowledge dissemination but also attracts greater citation rates [42]. Additionally, it is evident that journals with high impact factors tend to garner more citations than those with lower impact factors [43,44]. It is primarily due to their perceived prestige and influence in the academic realm. High-impact-factor journals prioritize quality over quantity in research, attracting a broader readership and increasing the visibility of their content [45,46]. Researchers are more inclined to submit their work to and cite articles from journals with higher impact factors, as this enhances the visibility and impact of their own research.

Currently, AI is revolutionizing the healthcare landscape with advanced algorithms that significantly enhance physicians’ diagnostic capabilities [47,48]. Its transformative influence extends to the interpretation of complex clinical data, notably helping the diagnosis of kidney diseases at an early stage [49]. The predictive capabilities of AI models play a crucial role in effective healthcare management, facilitating the early identification of individuals at risk of developing kidney diseases [50,51] and enabling timely interventions [24,52]. Our findings show that random forest and artificial neural networks are commonly used algorithms for disease classification and prediction. Additionally, AI models are helping to develop personalized treatment strategies by tailoring interventions to individual patient profiles, aiming for optimized outcomes while minimizing adverse effects [53]. AI tools also hold promising potential in the monitoring of drug prescriptions for kidney diseases [54]. Given the complex nature of these conditions and the intricate interplay between medications and renal function, AI systems can play a crucial role in ensuring optimal drug management [55,56,57]. By leveraging big data, AI may identify patterns and predict potential adverse reactions, enabling healthcare providers to monitor drug prescriptions to individual patients with CKD or those at risk of AKI [52,58]. However, the use of AI for drug monitoring and clinical care is in its infancy [59,60]. The transformative impact of AI in healthcare, particularly in the domain of kidney disease diagnosis, holds the promise of enhanced patient care, more effective treatments, and improved overall health outcomes [30,61,62].

Ensuring the safety of kidney disease patients when utilizing AI is crucial to effectively leverage its potential benefits. Physicians must implement robust tools and measures to protect patients from unexpected errors made by AI tools [63]. Firstly, AI algorithms must be trained and validated with diverse and representative datasets to enhance accuracy and prevent biases [64]. Health care providers should establish stringent regulatory frameworks and standards for AI in healthcare, ensuring strict adherence to ethical guidelines and legal requirements [65]. The implementation of fail-safe mechanisms and human oversight is essential, enabling clinicians to intervene and rectify any erroneous decisions [66]. Physicians should conduct regular audits of AI tools, ensure transparency in decision-making processes, and engage in open communication with patients regarding the role of AI tools. These actions are decisive in building trust and addressing concerns, particularly within the context of kidney disease treatment. Healthcare policymakers should develop harmonized global frameworks to address patients’ privacy regarding patient data analysis. It is important to navigate the diverse legal landscapes, particularly between Europe and the USA, and develop robust anonymization techniques to ensure mitigation of privacy risks. Given the concern, developing clear and standardized guidelines for data sharing and processing across borders is pivotal, fostering trust and compliance with varying international regulations [67].

It is essential for healthcare providers to establish robust data security measures, protecting sensitive information and preserving privacy. The design of AI algorithms should prioritize accuracy and minimize biases by incorporating diverse and representative datasets during training [68]. Ensuring transparency in the decision-making process, including providing clear explanations of how conclusions are reached, remains crucial [69]. Additionally, strict adherence to ethical guidelines and legal requirements, such as data protection laws, is imperative to uphold integrity. AI tools should be monitored continuously to ensure their adaptability to evolving datasets and changing circumstances.

This bibliometric study on the application of AI in kidney disease has yielded novel and insightful findings. Our study has not only highlighted the increasing significance of AI in the field of kidney disease but has also revealed emerging trends, key contributors, and the evolution of research over time. Our findings have provided valuable insights into the specific areas within kidney disease research where AI has made substantial contributions, shedding light on the potential for enhanced diagnostic, prognostic, and therapeutic applications. These novel findings contribute to a deeper understanding of the intersection between AI and kidney disease, offering a foundation for future research directions and the continued advancement of AI technologies in the realm of renal health.

### 4.1. Strengths and Limitations

This study has several strengths. This is the first bibliometric study offering a robust method for evaluating the application of AI in kidney disease research, providing valuable insights into the strengths and trends within this dynamic field. Moreover, this study provides a broad overview of the existing research landscape, highlighting key trends, potential contributions, and emerging themes in the field. Indeed, this study provides a comprehensive understanding of the current state of AI in kidney disease, allowing researchers and policymakers to pinpoint areas of high impact and innovation. There are several limitations in our study that require acknowledgment. First, data for this study was collected from a single database, although it is a standard database for conducting bibliometric analyses in health research [70,71,72]. This database contains all relevant variables and includes high quality peer-reviewed journals. Second, it is possible that our search terms may have excluded certain articles; nevertheless, our search strategy was developed through discussions with other experts and a comprehensive review of previously published articles. Third, while we have presented the top 10 most highly cited articles, it is important to note that articles published earlier are likely to receive more citations. Unfortunately, we encountered challenges in presenting a standardized approach for evaluating highly cited articles on a yearly basis. Additionally, we were unable to provide a citation rate stratified by journals and publication date, which could have offered more precise insights into whether journals with higher impact factors tend to garner more citations. Lastly, our study only focused on articles published in English, meaning there is a potential oversight of valuable contributions to the topic published in other languages.

### 4.2. Future Directions

AI holds significant promise in transforming the management of kidney disease in the future through various applications and advancements. Here are several ways in which AI can contribute to kidney disease management (Table 7):

While the integration of AI in kidney disease management presents promising opportunities, it is essential to address challenges related to data privacy, ethical considerations, and the need for collaboration between healthcare professionals and AI systems to ensure safe and effective implementation.

## 5. Conclusions

This bibliometric study comprehensively examined the impact of AI on kidney disease research, providing a comprehensive overview of the field’s evolution. Through bibliometric analysis, this study identified key themes, influential authors, high-impact journals, and the most productive countries that have shaped the discourse on AI in kidney disease research. This bibliometric analysis contributes to a nuanced understanding of the progress and focal points in the field, guiding future research endeavors and fostering collaboration in the pursuit of innovative solutions for kidney disease care.

## Figures and Tables

**Table 1:** The distribution of articles by year between 2010 and 2023.

Year	Number	Percentage	Annual Growth	Annual Growth Rate
2010	1	0.16	1	0.00
2011	4	0.63	3	300.00
2012	0	0.00	0	−100.00
2013	6	0.95	6	600.00
2014	3	0.48	−3	−50.00
2015	12	1.90	9	300.00
2016	11	1.74	−1	−8.33
2017	24	3.80	13	118.18
2018	30	4.75	6	25.00
2019	56	8.87	26	86.67
2020	73	11.57	17	30.36
2021	104	16.48	31	42.47
2022	172	27.26	68	65.38
2023	135	21.39	37	−21.51

**Table 2:** Top 10 journals published papers in the application of artificial intelligence on kidney disease research.

Rank	Journal	Country	Category	Record Count	% of 631	IF
1	Nephrology Dialysis Transplantation	England	Urology and nephrology	26	4.12	6.1
2	American Journal of Transplantation	Denmark	Transplantation	19	3.01	8.7
3	Scientific Reports	England	Multidisciplinary	17	2.69	4.6
4	Frontiers in Medicine	Switzerland	Medicine	11	1.74	3.9
5	IEEE Access	USA	Computer science	11	1.74	3.9
6	Journal of Clinical Medicine	Switzerland	Medicine	11	1.74	3.9
7	PLOS One	USA	Multidisciplinary	11	1.74	3.7
8	American Journal of Kidney Diseases	USA	Urology and nephrology	9	1.43	13.2
9	Diagnostics	Switzerland	Medicine	9	1.43	3.6
10	Computer in Biology and Medicine	USA	Computer science and interdisciplinary application	8	1.26	7.7

**Table 3:** Top 10 regions/countries publishing papers on the application of artificial intelligence in kidney disease research between 2010 and 2023.

Rank	Country	Number	Percentage
1	USA	164	25.99
2	People’s Republic of China	156	24.72
3	India	62	9.83
4	England	38	6.02
5	Italy	34	5.39
6	South Korea	34	5.39
7	Canada	27	4.28
8	Germany	27	4.28
9	Spain	26	4.12
10	Taiwan	26	4.12

**Table 4:** Top 10 institutes publishing papers on the application of artificial intelligence in kidney disease research between 2010 and 2023.

Rank	Institutions	Number	Percentage
1	Mayo Clinic	27	4.27
2	Harvard University	19	3.01
3	Sun Yat-Sen University	16	2.53
4	Sichuan University	15	2.37
5	University of California System	15	2.37
6	Harvard Medical School	14	2.21
7	Johns Hopkins University	13	2.06
8	Mayo Clinic Phoenix	13	2.06
9	Thammasat University	13	2.06
10	Massachusetts General Hospital	12	1.90

**Table 5:** Top 10 highly productive authors who published articles on the application of artificial intelligence in kidney disease research between 2010 and 2023.

Rank	Author	Number	Percentage
1	Cheungpasitporn W	16	2.54
2	Thongprayoon C	16	2.54
3	Leeaphorn N	13	2.06
4	Cooper M	12	1.90
5	Jadlowiec CC	12	1.90
6	Mao MA	12	1.90
7	Pattharanitima P	12	1.90
8	Kaewput W	11	1.74
9	Mao SA	11	1.74
10	Vaitla P	9	1.43

**Table 6:** Top 10 most cited published papers on the application of artificial intelligence in kidney disease research between 2010 and 2023.

Rank	Titles	Citations
1 [20]	Neural network and support vector machine for the prediction of chronic kidney disease: A comparative study. Computers in biology and medicine. 2019 Jun 1; 109:101–11	104
2 [21]	Diagnosis of chronic kidney disease based on support vector machine by feature selection methods. Journal of medical systems. 2017 Apr; 41:1–1.	88
3 [22]	A deep learning algorithm to detect chronic kidney disease from retinal photographs in community-based populations. The Lancet Digital Health. 2020 Jun 1;2(6): e295–302.	77
4 [23]	Comparison and development of machine learning tools in the prediction of chronic kidney disease progression. Journal of translational medicine. 2019 Dec;17(1):1–3.	77
5 [24]	Deep-learning models for the detection and incidence prediction of chronic kidney disease and type 2 diabetes from retinal fundus images. Nature biomedical engineering. 2021 Jun;5(6):533–45.	72
6 [25]	A machine learning methodology for diagnosing chronic kidney disease. IEEE Access. 2019 Dec 30; 8:20991–1002	59
7 [26]	Generating automated kidney transplant biopsy reports combining molecular measurements with ensembles of machine learning classifiers. American Journal of Transplantation. 2019 Oct 1;19(10):2719–31	57
8 [27]	A machine learning approach using survival statistics to predict graft survival in kidney transplant recipients: A multicenter cohort study. Scientific reports. 2017 Aug 21;7(1):8904.	57
9 [28]	Development of an artificial intelligence model to guide the management of blood pressure, fluid volume, and dialysis dose in end-stage kidney disease patients: proof of concept and first clinical assessment. Kidney diseases. 2019 Feb 1;5(1):28–33.	46
10 [29]	Detection and diagnosis of chronic kidney disease using deep learning-based heterogeneous modified artificial neural network. Future Generation Computer Systems. 2020 Oct 1; 111:17–26.	46

**Table 7:** Future applications of AI for kidney disease management.

Keyways	Process
Early detection and diagnosis	To detect patterns and early signs of kidney disease.
	To identify subtle biomarkers and risk factors that may not be immediately apparent to human clinicians.
Personalized treatment plans	To develop personalized treatment plans by considering individual patient data, including genetics, lifestyle, and treatment response.
	To anticipate disease progression and tailor interventions accordingly
Optimizing medication management	To enhance medication adherence by providing reminders, monitoring side effects, and adjusting dosages based on real-time patient data
	To identify patients at higher risk of nonadherence and enable proactive interventions.
Remote patient monitoring	To continuously monitor vital signs and other relevant health parameters, enabling remote patient monitoring.
Predictive analytics for complications	To predict complications associated with kidney disease, such as acute kidney injury, allowing for early intervention and prevention.
Efficient resource allocation	To optimize resource allocation by predicting patient admission rates, identifying high-risk populations, and allocating resources accordingly.

## Data Availability

Not applicable.

## Supplementary Materials

The following supporting information can be downloaded at: https://www.mdpi.com/article/10.3390/diagnostics14040397/s1, Table S1: Search keywords.
