# Supplementary material for: Artificial Intelligence in Kidney Disease: A Comprehensive Study and Directions for Future Research
Source: Diagnostics (Basel). 2024 Feb 12;14(4):397. doi: 10.3390/diagnostics14040397 (PMC10887584; doi:10.3390/diagnostics14040397)
Supplement: Supplementary file 1 [file diagnostics-14-00397-s001.zip › diagnostics-2780678-supplementary.pdf]

Table S1: Search keywords.

Topic	Keywords
Artificial intelligence	artificial intelligence OR "computational intelligence" OR "deep learning" OR "Computer-aided" OR "machine learning" OR "Support vector machine" OR "data learning" OR " artificial neural network" OR "digital image" OR "convolutional neural network" OR "evolutionary algorithms" OR "feature learning" OR "reinforcement learning" OR "Big data" OR "image segmentation" OR "Hybrid intelligent system" OR "hybrid intelligent system" OR "recurrent neural network" OR "natural language processing" OR "Bayesian network " OR "Bayesian learning" OR " random forest" OR "Evolutionary algorithms" OR "multiagent system"
Kidney diseases	“chronic kidney failure” OR “chronic renal insufficiency” OR “chronic kidney disease*” OR “renal failure*” OR “kidney failure” OR “renal impairment” OR “kidney impairment” OR “kidney dysfunction” OR “renal dysfunction” OR “reduced renal function” OR “CKD” OR “progressive kidney” OR “glomerular filtration rate” OR “GFR” OR “eGFR” OR proteinuri* OR “albuminuria” OR “microalbuminuria” OR “end-stage renal disease” OR “ESRD” OR “end-stage kidney disease” OR “ESKD” OR “dialysis” OR “renal replacement therapy” OR “kidney transplant” OR “lupus nephritis” OR “acute kidney injuries” OR “acute renal injury” OR “acute renal injuries” OR “acute renal insufficiencies” OR “acute kidney insufficiencies” OR “acute kidney insufficiency” OR “acute renal failure” OR “acute renal failures” OR “AKI” OR “systemic lupus erythematosus” OR “SLE” OR “nephrotic syndrome” OR “NS” OR “glomerulonephritides” OR “iga glomerulonephritis” OR “iga nephropathy” OR “nephritis”
